# Supplementary material for: Asymmetric reconstruction of mammalian reovirus reveals interactions among RNA, transcriptional factor µ2 and capsid proteins
Source: Nat Commun. 2021 Jul 7;12:4176. doi: 10.1038/s41467-021-24455-4 (PMC8263624; doi:10.1038/s41467-021-24455-4)
Supplement: Supplementary file 1 — Supplementary Information [file 41467_2021_24455_MOESM1_ESM.pdf]

Asymmetric reconstruction of mammalian reovirus reveals interactions among RNA, transcriptional factor μ2 and capsid proteins

Muchen Pan, Ana L. Alvarez-Cabrera, Joon S. Kang, Lihua Wang, Chunhai Fan, Z. Hong Zhou*

Supplementary Information

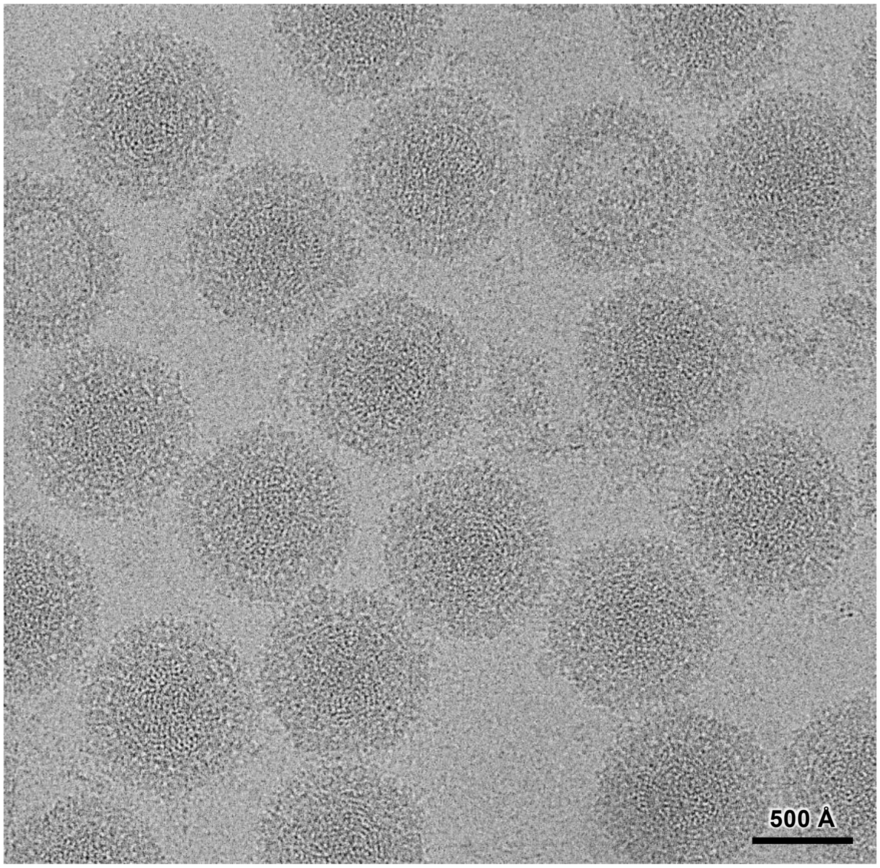

Supplementary Figure 1 CryoEM micrograph of MRV ISVP particles.

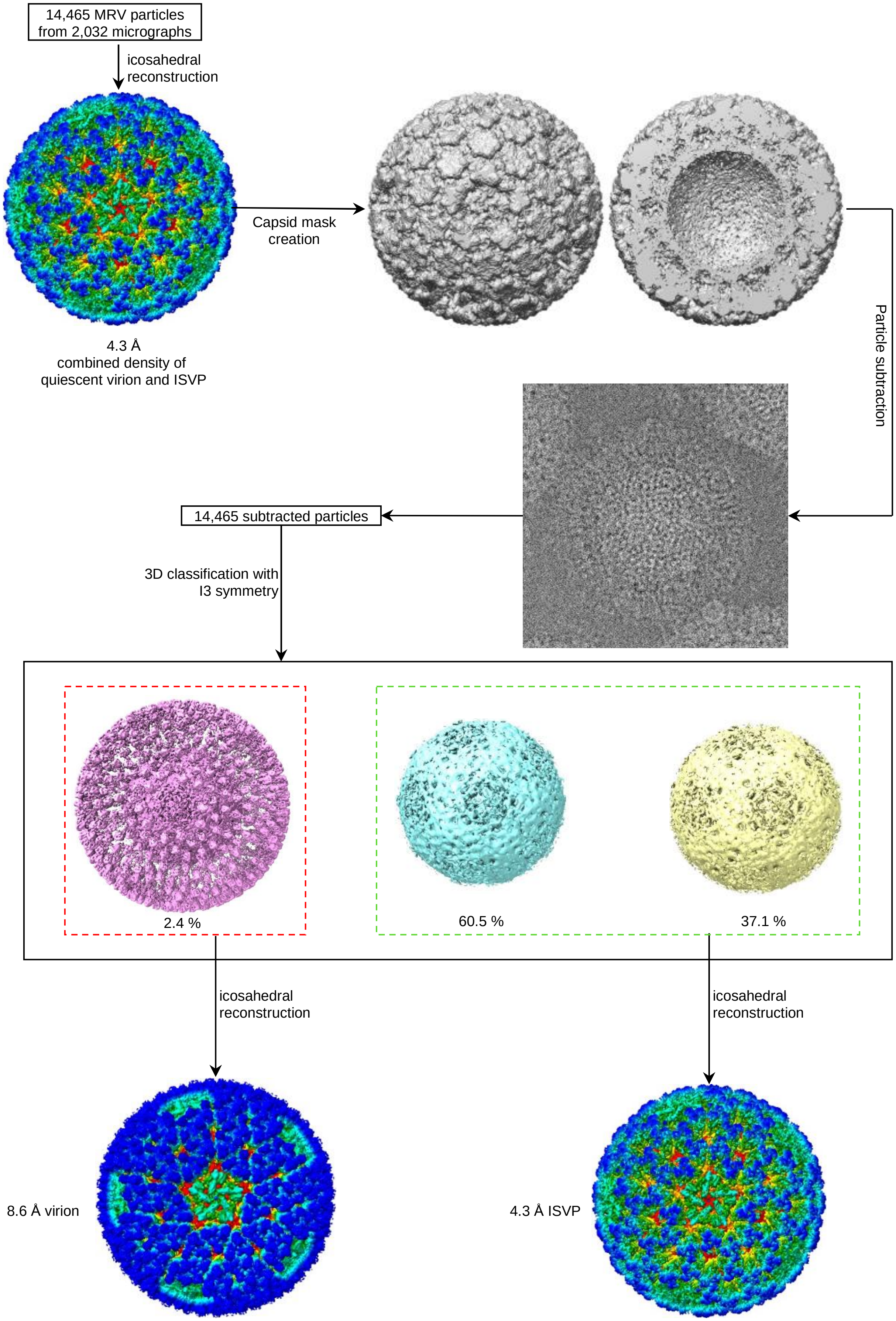

Supplementary Figure 2 Separation of MRV ISVP and quiescent virion. Subtraction and 3D classification workflow for classifying and separating ISVP and virion particles. Arrows with associated text denote data-processing steps. Boxed text describes the properties of the data.

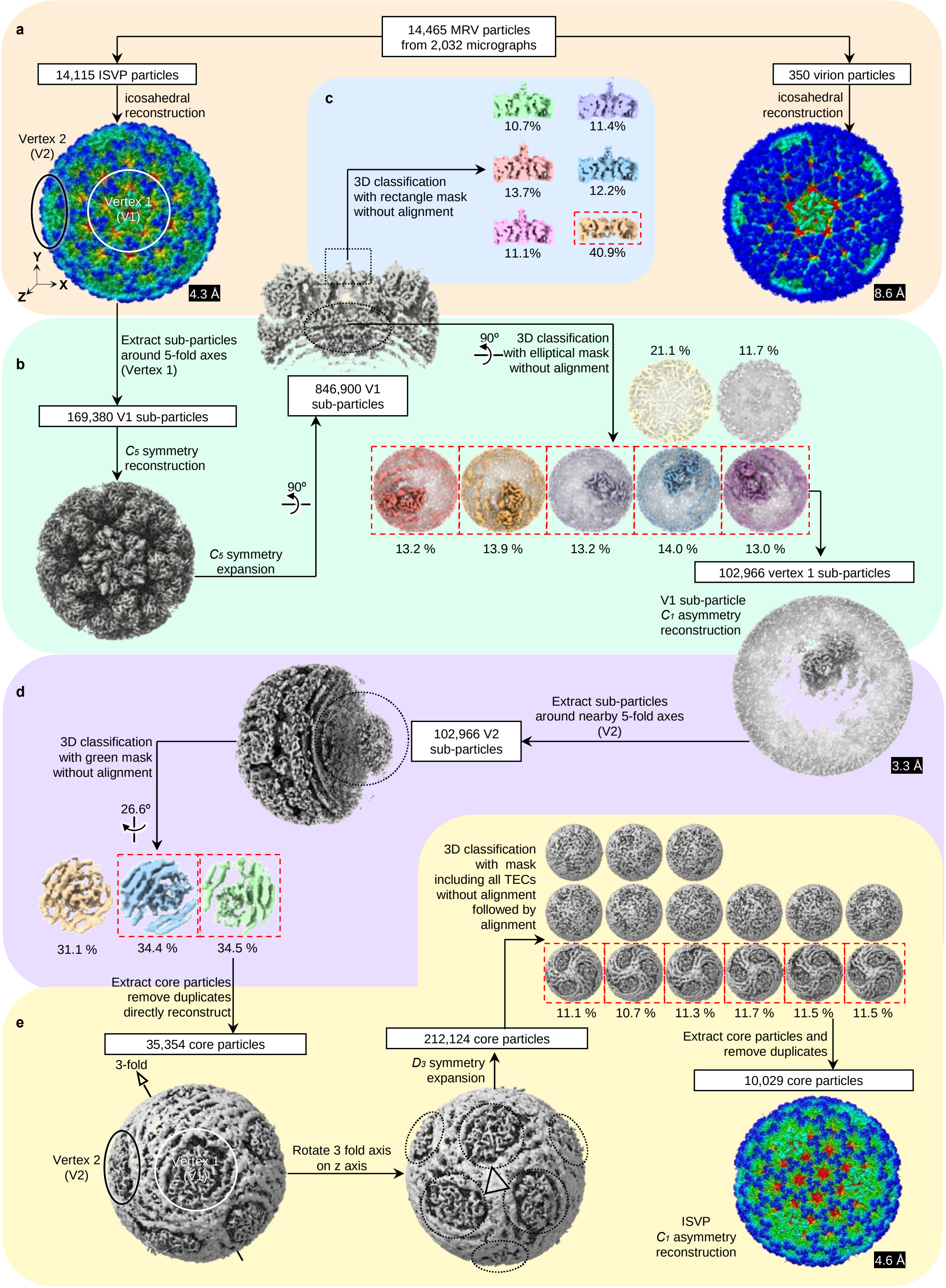

Supplementary Figure 3 Data processing workflow for the MRV ISVP reconstruction. Arrows with associated text denote data-processing steps. Boxed text describes the properties of the data. **a** 3D classification separating virions and ISVP (following Supplementary Figure 1). **b, c** Symmetry expansion from icosahedral reconstruction (I3) to vertex sub-particle reconstruction (see Methods section Vertex sub-particle reconstruction). When the boxed sub-particles are from the inner capsid region (**b**), this step yielded five nearly identical asymmetric sub-particle reconstructions with TEC (V1), one reconstruction without TEC and one junk reconstruction. When the boxed sub-particles are from the outer capsid region (**c**), this step yielded four reconstructions with σ1 spike and one without. **d** Boxing the immediately adjacent vertex sub-particles

(V2) for a second round of sub-particle C_1 classification and reconstruction, yielding a STAR file to be used for D_{3d} symmetry reconstruction of ISVP (see Methods section D_{3d}-symmetry reconstruction of the ISVP core). **e** Steps for decoupling D_{3d} symmetry to C_1 asymmetric reconstruction for the ISVP. The result is six nearly identical asymmetrical ISVP reconstruction with ten TEC-containing vertices and two TEC-absent vertices as shown in Figure 1 [see Methods section Decoupling D_{3d} symmetry for asymmetric (C_1) reconstruction].

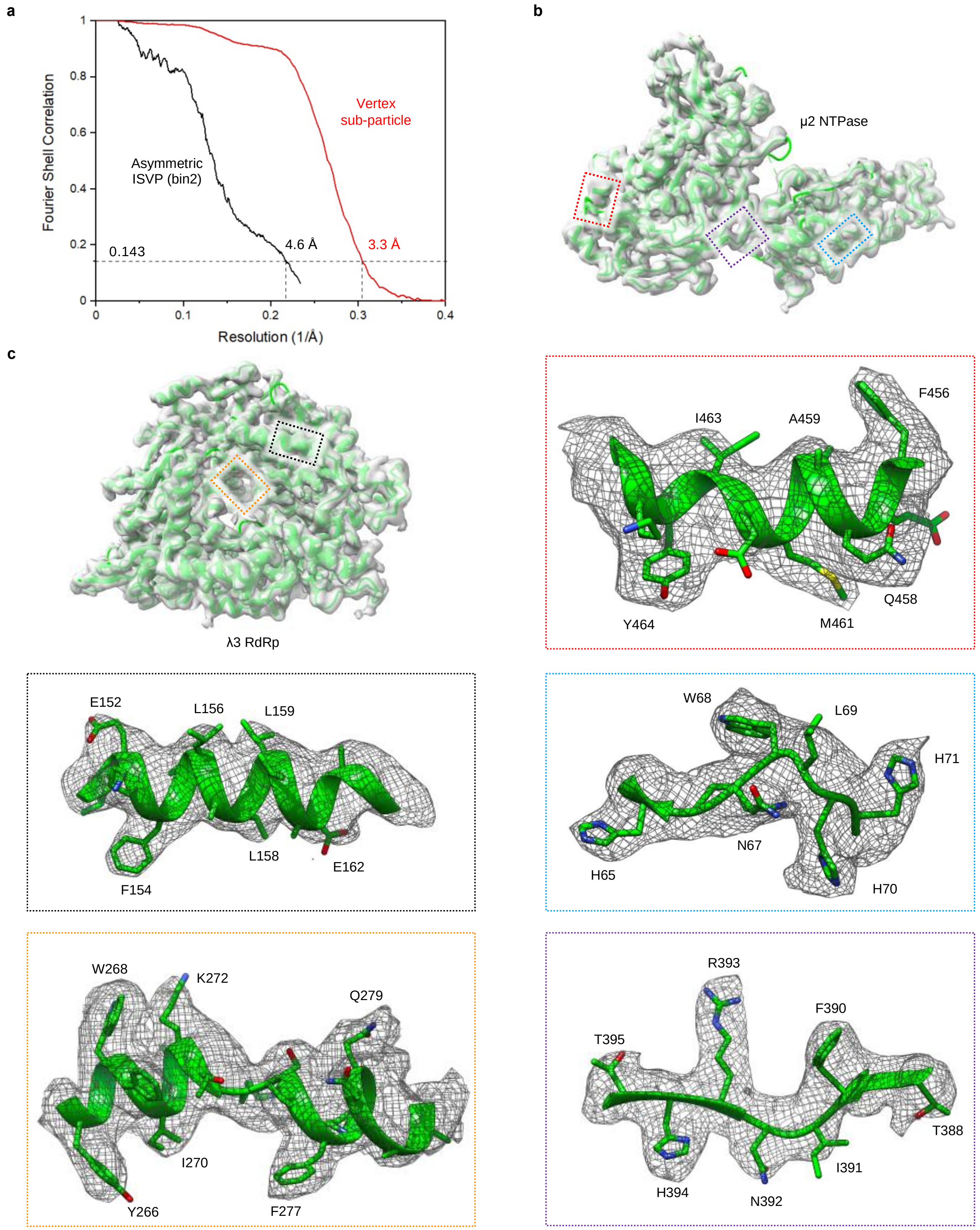

Supplementary Figure 4 Resolution verification. **a** Fourier shell correction resolution evaluation for vertex sub-particle and asymmetric ISVP. **b**, **c** Superposition of Density map (gray mesh) and atomic model (green sticks and ribbons) of α helixes and β strands in NTPase and RdRp, showing side chain densities of similar quality. Zoom-in views from the boxes with same edge color, respectively.

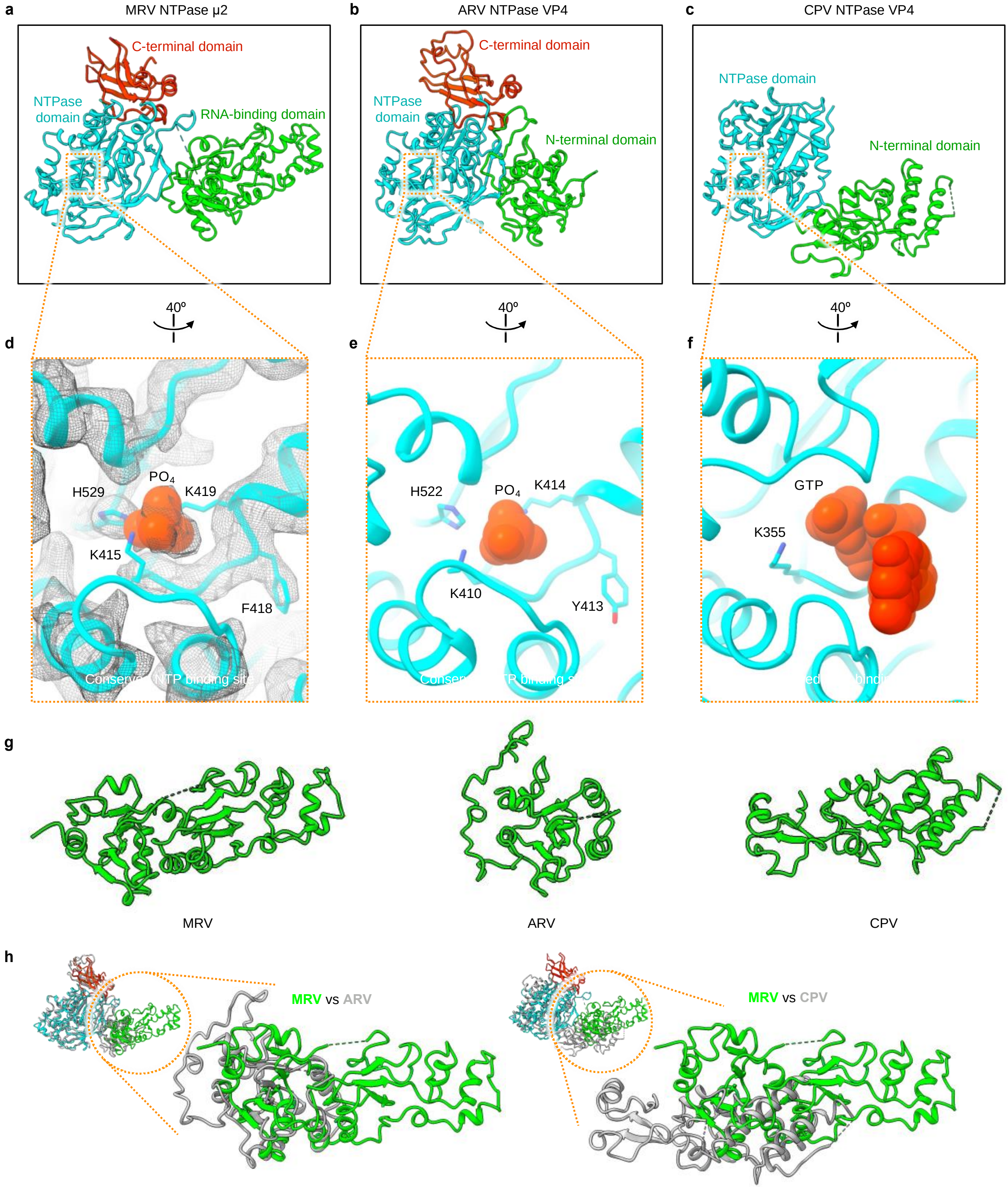

Supplementary Figure 5 Comparison of the NTPase homologs in MRV, ARV, and CPV. **a-c** Ribbon representation of NTPase proteins of MRV μ2 (**a**), ARV VP4 (**b**), and CPV VP4 (**c**). The domains are colored as in Fig. 2**b**. **d-f** Zoom-in view of orange-boxed regions in (**a-c**), detailing the conserved NTP binding site of MRV (**d**), ARV (**e**), and CPV (**f**). The density is shown in mesh style (**d**). The binding residues are shown in stick style and phosphate groups/NTP are shown in red and ball style. **g**, **h** Superimposing of NTPase proteins of MRV vs ARV and MRV vs CPV, highlighting the RNA-binding domain of MRV and N-terminal domain of ARV, CPV and their superpositions.

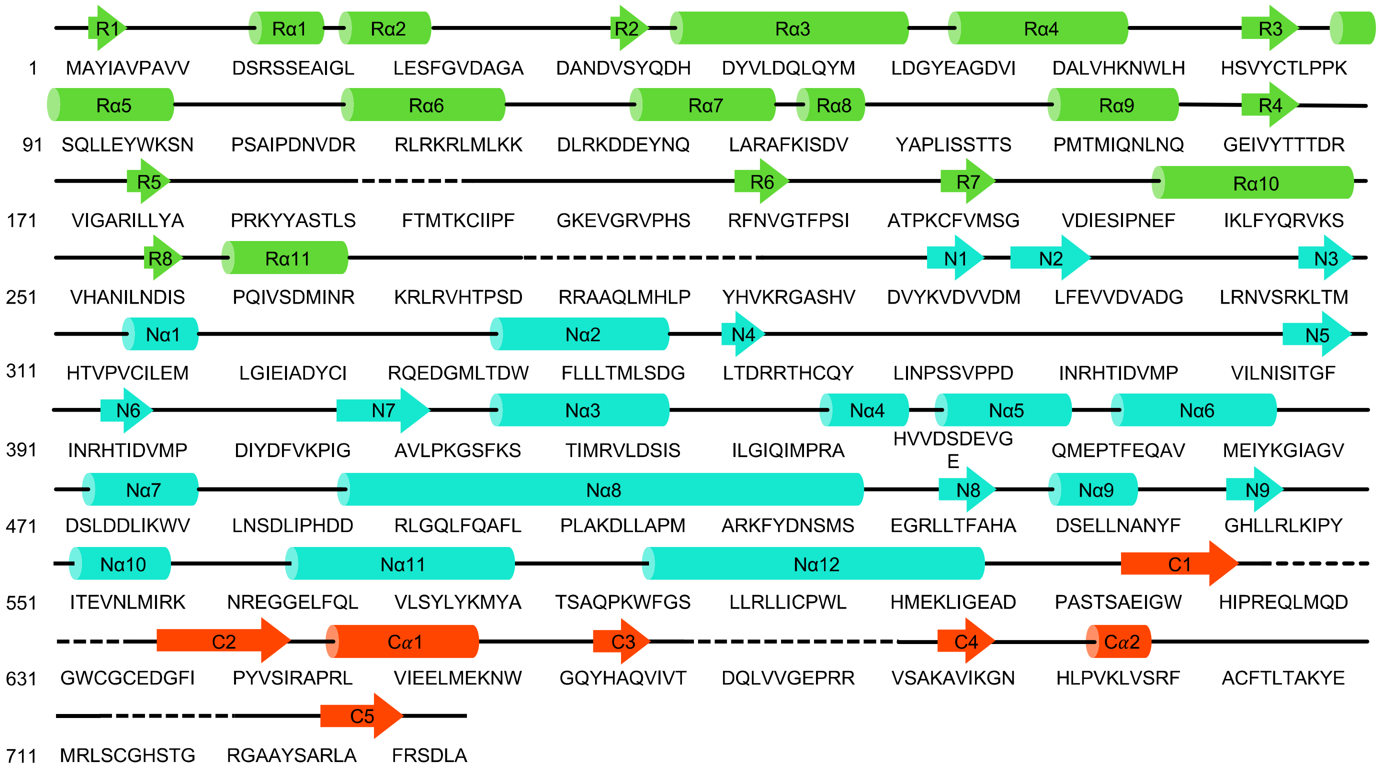

Supplementary Figure 6 Sequence and secondary structure assignment of MRV NTPase μ2. The amino acid sequence of μ2 with secondary-structure assignments indicated above the corresponding sequences. Helices, strands, and coils/turns are shown in rods, arrows, and black lines, respectively. Helix and strand numbers are preceded by R, N, or C to indicate RNA-binding domain, NTPase domain and C-terminal domain, respectively. Dashed lines indicate regions not modeled. The domains are colored as in Fig. 2**b**.

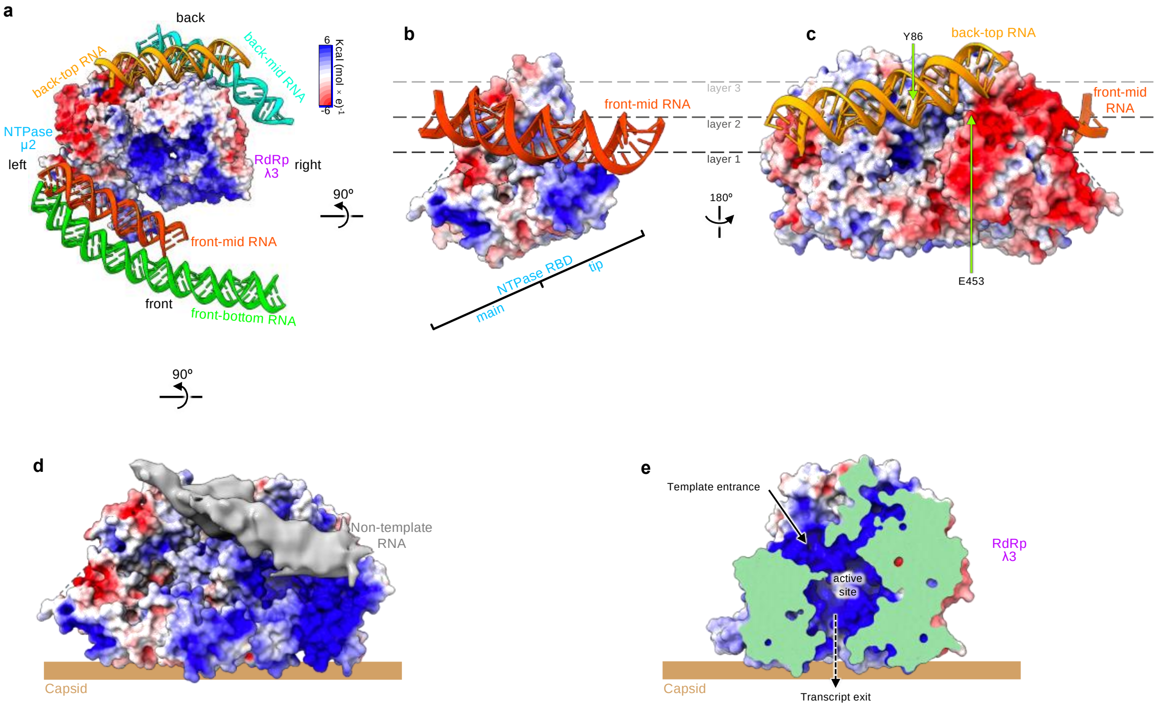

Supplementary Figure 7 RNA and TEC electrostatic potentials. **a** Surface representation of TEC model with positive, neutral and negative electrostatic potentials indicated in blue, white, and red, respectively. And ribbon representation of “front-bottom”, “front-mid”, “back-mid”, and “back-top” RNA duplex, colored as Fig. 4. **b** Ribbon representation of “front-mid” RNA and surface representation of NTPase μ2 electrostatic potentials, the 90º rotated view of (**a**). **c** Ribbon representation of “back-top” RNA and surface representation of TEC electrostatic potentials, the 180º rotated view of (**a**). The green arrow indicating the anchoring and repulsion sites. **d** Density of terminal RNA (gray) and surface representation of TEC electrostatic potentials. **e** Cut-open view of RdRp surface, highlighting the positively charged template entrance, RdRp active site, and transcript exit.

Supplementary Table 1 CryoEM data collection, refinement and validation statistics

	C1 Vertex sub-particle TEC and λ1 (EMD-31183) (PDB-7ELH)	C1 Vertex sub-particle μ1 and λ2 (EMD-31184) (PDB-7ELL)	Asymmetric ISVP (EMD-31187)	Icosahedral virion (EMD-31188)
**Data collection and processing**				
Magnification	130,000	130,000	130,000	130,000
Voltage (kV)	300	300	300	300
Electron exposure (e^- per Å2)	~56	~56	~56	~56
Defocus range (μm)	-0.5 to -2.8	-0.5 to -2.8	-0.5 to -2.8	-0.5 to -2.8
Pixel size (Å)	1.07	1.07	2.14	4.28
Symmetry imposed	C_1	C_1	C_1	I3
Initial particle images (no.)	846,900	846,900	14,465	14,465
Final particle images (no.)	102,966	61,861	10,029	350
Map resolution (Å)	3.3	3.8	4.6	8.6
FSC threshold	0.143	0.143	0.143	0.143
**Refinement**				
Initial model used (PDB code)	1MUK, 6M99	2CSE, 1EJ6		
Model resolution (Å)	3.5	3.1		
FSC threshold	0.143	0.143		
Map sharpening *B* factor ($Å^2$)	-130.0	-129.5		
Model composition				
Non-hydrogen atoms	111,926	60,578		
Protein residues	13,381	7,919		
RNA/DNA Nucleotides	310	0		
Ligands (MYR)	0	10		
R.M.S. deviations				
Bond lengths (Å)	0.007	0.007		
Bond angle (º)	1.109	1.116		
Validation				
MolProbity score	1.74	2.23		
Clash score	8.93	16.21		
Poor rotamers (%)	0.09	0.13		
Ramachandran plot				
Favored (%)	96.09	90.97		
Allowed (%)	3.86	8.96		
Disallowed (%)	0.05	0.08		

Supplementary Table 2 Comparison of genomic segments and encoded proteins in MRV, ARV and CPV

	MRV[17]			ARV[36]			CPV[37]		
**Protein functions**	Genomic RNA segment name	Protein name	Protein length (a.a.)	Genomic RNA segment No.	Protein name	Protein length (a.a.)	Genomic RNA segment No.	Protein name	Protein length (a.a.)
RNA polymerase	L1	λ3	1267	2	VP2	1259	2	RdRp (V2)	1226
mRNA capping/turret protein	L2	λ2	1289	1	VP1 (TP)	1299	4	TP (V3)	1058
Capsid shell protein (CSP)	L3	λ1	1275	3	VP3 (CSP)	1214	1	CSP (V1)[59]	1333
NTPase	M1	μ2	736	5	VP4	728	6	NTPase (V4)	561
Membrane penetration protein	M2	μ1	708	6	VP5	648			
Microtubule-binding at viral factory	M3	μNS	721	4	NS1	742			
Receptor binding protein	S1	σ1	455	7	NS4,5	274,146			
Clamp protein	S2	σ2	418	8	VP6	412	7	LPP (V5)	448
Binding to μNS at viral factory	S3	σNS	366	9	NS2	352			
Protection protein	S4	σ3	365	10	VP7	276			
				11	NS3	244			
Receptor binding and membrane penetration							3	Spike A (VP3)	1239
							5	NSP5 (VP5)	881
							8	NSP8 (p44)	390
							9	NSP9 (NS5)	320
Polyhedra formation, virus protection							10	Poly-hedrin	248

Abbreviations: L: large; M: medium; S: small; TP: turret protein; CSP: capsid shell protein; LPP: clamping/large protrusion protein; NS or NSP: non-structural protein

Reference

59. Hagiwara, K., Naitow, H. Assembly into single-shelled virus-like particles by major capsid protein VP1 encoded by genome segment S1 of Bombyx mori cypovirus 1. *J. Gen. Virol.* **84**, 2439-2441 (2003).
