# Supplementary material for: Asymmetric reconstruction of mammalian reovirus reveals interactions among RNA, transcriptional factor µ2 and capsid proteins
Source: Nat Commun. 2021 Jul 7;12:4176. doi: 10.1038/s41467-021-24455-4 (PMC8263624; doi:10.1038/s41467-021-24455-4)
Supplement: Supplementary file 3 — Description of Additional Supplementary Files [file 41467_2021_24455_MOESM3_ESM.pdf]

Description of Additional Supplementary Files

File name: Supplementary Movie 1.

Description: Shaded surface view of the asymmetrical reconstruction of MRV. Color coding is the same as in Fig. 1, highlighting TEC [RdRp (purple) & NTPase (blue)], and dsRNA (gray) densities. Related to Fig. 1.

File name: Supplementary Movie 2.

Description: Location and structural details of TEC. The animation starts with the sub-particle reconstruction showing as shaded surface and ends with ribbon diagram of the TEC atomic model, colored by domains of RdRp λ3 and NTPase μ2. Related to and colored as in Fig. 2.

File name: Supplementary Movie 3.

Description: Genomic dsRNA surrounding TEC. The animation starts as in Supplementary Movie 2 but highlights TEC [RdRp (purple) & NTPase (blue)], “front-bottom” RNA (green), “front-mid” RNA (red), “back-mid” RNA (cyan), and “back-top” RNA (yellow). Related to and colored as in Fig. 4.

File name: Supplementary Movie 4.

Description: RNA structures around the active site of the RdRp. The animation starts as in Supplementary Movie 2 but highlights the terminal RNA branching into its non-template strand and template (orange), and the putative transcript density(blue). Related to and colored as in Fig. 4.

File name: Supplementary Movie 5.

Description: Crystal structure of σ1 fitted into the cryoEM subparticle reconstruction. The animation starts as in Supplementary Movie 2 but highlights the σ1 crystal structure (red) fitted into the σ1 cryoEM density (gray) displayed at a relatively low density threshold.
