# Supplementary material for: Asymmetric reconstruction of mammalian reovirus reveals interactions among RNA, transcriptional factor µ2 and capsid proteins
Source: Nat Commun. 2021 Jul 7;12:4176. doi: 10.1038/s41467-021-24455-4 (PMC8263624; doi:10.1038/s41467-021-24455-4)
Supplement: Supplementary file 9 — Reporting summary [file 41467_2021_24455_MOESM9_ESM.pdf]

nature research

Corresponding author(s): Z.Hong Zhou

Last updated by author(s): Jun 2, 2021

Reporting Summary

Nature Research wishes to improve the reproducibility of the work that we publish. This form provides structure for consistency and transparency in reporting. For further information on Nature Research policies, see our Editorial Policies and the Editorial Policy Checklist.

Statistics

For all statistical analyses, confirm that the following items are present in the figure legend, table legend, main text, or Methods section.

n/a	Confirmed	
☒	☐	The exact sample size (n) for each experimental group/condition, given as a discrete number and unit of measurement
☒	☐	A statement on whether measurements were taken from distinct samples or whether the same sample was measured repeatedly
☒	☐	The statistical test(s) used AND whether they are one- or two-sided<br>*Only common tests should be described solely by name; describe more complex techniques in the Methods section.*
☒	☐	A description of all covariates tested
☒	☐	A description of any assumptions or corrections, such as tests of normality and adjustment for multiple comparisons
☒	☐	A full description of the statistical parameters including central tendency (e.g. means) or other basic estimates (e.g. regression coefficient) AND variation (e.g. standard deviation) or associated estimates of uncertainty (e.g. confidence intervals)
☒	☐	For null hypothesis testing, the test statistic (e.g. F, t, r) with confidence intervals, effect sizes, degrees of freedom and P value noted<br>*Give P values as exact values whenever suitable.*
☒	☐	For Bayesian analysis, information on the choice of priors and Markov chain Monte Carlo settings
☒	☐	For hierarchical and complex designs, identification of the appropriate level for tests and full reporting of outcomes
☒	☐	Estimates of effect sizes (e.g. Cohen's d, Pearson's r), indicating how they were calculated

Our web collection on statistics for biologists contains articles on many of the points above.

Software and code

Policy information about availability of computer code

Data collection	SerialEM3.8
Data analysis	EMAN2, Ethan, MotionCor 2, CTFFIND 4, RELION 3.1, USFC ChimeraX1.1.1, COOT0.8.9.2, PHENIX1.18.2, PDB2PQR2.1.1, APBS1.5

For manuscripts utilizing custom algorithms or software that are central to the research but not yet described in published literature, software must be made available to editors and reviewers. We strongly encourage code deposition in a community repository (e.g. GitHub). See the Nature Research guidelines for submitting code & software for further information.

Data

Policy information about availability of data

All manuscripts must include a data availability statement. This statement should provide the following information, where applicable:

- Accession codes, unique identifiers, or web links for publicly available datasets
- A list of figures that have associated raw data
- A description of any restrictions on data availability

C1 Vertex sub-particle TEC and λ1 (EMD-31183, PDB-7ELH); C1 Vertex sub-particle μ1 and λ2 (EMD-31184, PDB-7ELL); Asymmetric ISVP (EMD-31187); Icosahedral virion (EMD-31188); Asymmetric σ1 density (EMD-31220)

Field-specific reporting

Please select the one below that is the best fit for your research. If you are not sure, read the appropriate sections before making your selection.

☒ Life sciences ☐ Behavioural & social sciences ☐ Ecological, evolutionary & environmental sciences

For a reference copy of the document with all sections, see nature.com/documents/nr-reporting-summary-flat.pdf

Life sciences study design

All studies must disclose on these points even when the disclosure is negative.

Sample size	Sample sizes were estimated on the basis of previous study (PMID 26503045).
Data exclusions	For cryo-EM analysis, particles that do not belong to the class of interest or have poor qualities based on well established cryo-EM principle were excluded after rounds of 2D and 3D classification. This is standard practice required to obtain high resolution cryo-EM structure of the class of interest.
Replication	Reconstructions are the result of calculations performed on the collected data set. Therefore, all reconstructions are reproducible if the workflow is maintained.
Randomization	No grouping required for our studies.
Blinding	Blinding was not necessary for structure determination by single-particle cryoEM method.<br>Since there is no grouping required for our studies, there is no blinding with respect to grouping.

Reporting for specific materials, systems and methods

We require information from authors about some types of materials, experimental systems and methods used in many studies. Here, indicate whether each material, system or method listed is relevant to your study. If you are not sure if a list item applies to your research, read the appropriate section before selecting a response.

Materials & experimental systems

n/a	Involved in the study	
☒	☐	Antibodies
☒	☐	Eukaryotic cell lines
☒	☐	Palaeontology and archaeology
☒	☐	Animals and other organisms
☒	☐	Human research participants
☒	☐	Clinical data
☒	☐	Dual use research of concern

Methods

n/a	Involved in the study	
☒	☐	ChIP-seq
☒	☐	Flow cytometry
☒	☐	MRI-based neuroimaging
